# The mevalonate precursor enzyme HMGCS1 is a novel marker and key mediator of cancer stem cell enrichment in luminal and basal models of breast cancer

**DOI:** 10.1371/journal.pone.0236187

**Published:** 2020-07-21

**Authors:** Claire A. Walsh, Nina Akrap, Elena Garre, Ylva Magnusson, Hannah Harrison, Daniel Andersson, Emma Jonasson, Svanheidur Rafnsdottir, Hani Choudhry, Francesca Buffa, Jiannis Ragoussis, Anders Ståhlberg, Adrian Harris, Göran Landberg

**Affiliations:** 1 Sahlgrenska Cancer Center, Department of Laboratory Medicine, Gothenburg, Sweden; 2 Paterson Institute for Cancer Research, The Christie NHS Foundation Trust, Manchester, United Kingdom; 3 Department of Surgery, Sahlgrenska University Hospital, Gothenburg, Sweden; 4 Department of Biochemistry, Faculty of Science, King Abdulaziz University, Jeddah, Saudi Arabia; 5 The Weatherall Institute of Molecular Medicine, University of Oxford, John Radcliffe Hospital, Headington, Oxford, United Kingdom; 6 McGill University and Genome Quebec Innovation Centre, Montreal, Canada; 7 Wallenberg Centre for Molecular and Translational Medicine, University of Gothenburg, Gothenburg, Sweden; 8 Department of Clinical Genetics and Genomics, Sahlgrenska University Hospital, Gothenburg, Sweden; Qatar Biomedical Research Institute, QATAR

## Abstract

The definitive characterization of common cancer stem cell (CSCs) subpopulations in breast cancer subtypes with distinct genotypic and phenotypic features remains an ongoing challenge. In this study, we have used a non-biased genome wide screening approach to identify transcriptional networks that may be specific to the CSC subpopulations in both luminal and basal breast cancer subtypes. In depth studies of three CSC-enriched breast cancer cell lines representing various subtypes of breast cancer revealed a striking hyperactivation of the mevalonate metabolic pathway in comparison to control cells. The upregulation of metabolic networks is a key feature of tumour cells securing growth and proliferative capabilities and dysregulated mevalonate metabolism has been associated with tumour malignancy and cellular transformation in breast cancer. Furthermore, accumulating evidence suggests that Simvastatin therapy, a mevalonate pathway inhibitor, could affect breast cancer progression and reduce breast cancer recurrence. When detailing the mevalonate pathway in breast cancer using a single-cell qPCR, we identified the mevalonate precursor enzyme, HMGCS1, as a specific marker of CSC-enriched subpopulations within both luminal and basal tumour subtypes. Down-regulation of HMGCS1 also decreased the CSC fraction and function in various model systems, suggesting that HMGCS1 is essential for CSC-activities in breast cancer in general. These data was supported by strong associations between HMGCS1 expression and aggressive features, such as high tumour grade, p53 mutations as well as ER-negativity in lymph node positive breast cancer. Importantly, loss of HMGCS1 also had a much more pronounced effect on CSC-activities compared to treatment with standard doses of Simvastatin. Taken together, this study highlights HMGCS1 as a potential gatekeeper for dysregulated mevalonate metabolism important for CSC-features in both luminal and basal breast cancer subtypes. Pharmacological inhibition of HMGCS1 could therefore be a superior novel treatment approach for breast cancer patients via additional CSC blocking functions.

## Introduction

Breast cancer is a distinctly heterogeneous disease, characterized by a complex and dynamic tumour cell population and a highly plastic tumour microenvironment [1]. It is generally accepted that breast cancer tumour cells are organized in a hierarchical fashion and that small subpopulations of cancer stem cells (CSCs) are actively resident within all tumour subtypes. CSCs are endowed with the capacity for self-renewal and multi-lineage differentiation, tumourigenicity, invasiveness and therapeutic resistance, all features that facilitate tumour progression, disease recurrence, and metastasis [2–4]. Specific therapeutic targeting of small CSC subpopulations is challenging but could have profound and long lasting clinical benefits for patients. Several methods of functional enrichment for CSCs have been utilized to facilitate better understanding and characterization of these subpopulations [5–7]. As a result, numerous genes and cell surface markers have already been associated with CSC-like functional behaviour within various breast cancer subtypes [4, 8, 9], however, a common marker of CSC subpopulations remains to be clearly defined. The “reprogramming of energy metabolism” is a recognized hallmark of cancer, whereby tumour cells can adapt their cellular metabolism to satisfy the bioenergetic and biosynthetic requirements for sustained growth and proliferation [10–12]. Tumour cells undergo a metabolic switch from aerobic, oxidative metabolism to high levels of aerobic glycolysis [13]. As a result, the oxidative phosphorylation reaction remains incomplete and leads to increased export of acetyl-CoA from the mitochondria into the cytosol. Cytosolic acetyl-CoA molecules are building blocks for lipogenic and anabolic reactions to promote cell growth and proliferation [14]. In breast cancer, the mevalonate metabolic pathway for cholesterol biogenesis and protein prenylation has been implicated with tumour cell transformation, malignancy and the specific regulation of basal-derived CSCs [15–17]. Furthermore, pharmacological blockades of the mevalonate pathway using statin or nitrogen-containing bisphosphonate therapeutics reduced the self-renewal capacity of the basal-derived CSCs, tumour cell motility, osteolytic bone lesions and the risk of breast cancer recurrence [16, 18–20].

The purpose of this study was to utilize transcriptome-wide screening of CSC subpopulations to elucidate any signalling networks that may be actively up-regulated independent of explicit breast cancer subtypes. The mevalonate pathway was identified and single-cell gene expression profiling was applied to better characterize the pathway in models of luminal and basal breast cancer. This data highlighted the mevalonate precursor enzyme, 3-hydroxy-3-methylglutaryl-CoA synthase 1 (HMGCS1), whose up-regulation is a common transcriptional event in CSC-enriched subpopulations of breast cancer cell lines. HMGCS1 catalyses the chemical conversion of acetoacetyl-CoA present within the cytosol to 3-hydroxy-3-methylglutaryl-CoA (HMG-CoA), which is the chemical structure necessary for HMG-CoA to successfully enter and initiate the mevalonate pathway [14, 21]. HMGCS1 was further investigated as an independent entity and its effects on functional CSC-enrichment and CSC-associated genetic signatures were studied using transient knockdown systems and single-cell analysis. Importantly, in our experimental models, inhibition of HMGCS1 produced a more potent functional inhibition of CSC-associated activity than standard statin treatment. To the extent possible, this study sought to characterize the role of mevalonate metabolism and the specific key enzyme, HMGCS1, on specific CSC subpopulations, with the goal of better understanding the predominant transcriptional regulatory networks in these highly tumourigenic and elusive cells.

## Materials and methods

### Cell culture, *HMGCS1* knockdown and simvastatin treatment

MCF-7, T47D and MDA-MB-231 cell lines (ATCC, Manassas, VA, USA; HTB-22™, HTB-133™ and HTB-26™ respectively) were cultured in accordance with ATCC recommendations at 37°C in a 5% CO2 humidified atmosphere. MCF-7 was cultured in Dulbecco’s modified Eagle’s medium (DMEM) supplemented with 10% fetal bovine serum, 1% penicillin/streptomycin, 1% L-glutamine (all from Thermo Fisher Scientific) and 1% MEM Non-Essential Amino Acids (Sigma-Aldrich). MDA-MB-231 and T47D were cultured in RPMI-1640 medium supplemented with 10% fetal bovine serum, 1% penicillin/streptomycin, 1% sodium pyruvate and 1% L-glutamine (Thermo Fisher Scientific). Cell lines were confirmed as mycoplasma-free (Mycoplasma PCR Detection Kit, Applied Biological Materials Inc., Richmond, BC, Canada). *HMGCS1* gene expression was silenced with 30 nM *HMGCS1* siRNA or treated with Trilencer-27 Universal Scrambled Negative Control siRNA (Origene Technologies Inc., Rockville, MD, USA) for 72 hours using the Viromer Blue transfection system (Lipocalyx, Halle, Germany). Cells were treated with 1 μM Simvastatin (S6196, Sigma-Aldrich, St. Louis, MO, USA) or DMSO as vehicle control (Sigma-Aldrich, St. Louis, MO, USA) for 48 hours. Following *HMGCS1* gene silencing cells were grown in 16-hour and 5-day suspension cultures (see below).

### Cancer stem cell (CSC) enrichment methods

To enrich for CSCs, single cell suspensions were seeded in 1.2% w/v poly(2-hydroxyethyl methacrylate)/95% ethanol-coated plates (Sigma-Aldrich) and grown in phenol red free DMEM/F-12 (Life Technologies, Carlsbad, CA, USA) containing 2% B27 supplement (Life Technologies), 20 ng mL^-1^ EGF (BD Biosciences, Franklin Lakes, NJ, USA) and 1% penicillin/streptomycin (PAA Laboratories, Pasching, Austria) at a density of 500 cells cm^-2^. Anoikis-resistant cells and mammospheres were harvested after 16 hours or 5 days, respectively, as described [22, 23]. Manual viability counts of anoikis-resistant cells were conducted with Trypan Blue exclusion dye (Sigma-Aldrich).

### RNA sequencing and data analysis

For RNA sequencing total RNA was extracted using the RNeasy Mini Kit (Qiagen, Limburg, Netherlands) and treated with RNase-free DNase kit (Qiagen). Directional RNA-sequencing libraries were prepared using the ScriptSeq™ v2 RNA-Seq kit (Epicentre, Madison, WI, USA) according to the manufacturer’s protocol. Library abundance was assessed using the Qubit dsDNA HS Assay on the Qubit 2.0 fluorometer (both from Life Technologies). Libraries were sequenced on the Illumina HiSeq platform (Illumina, San Diego, CA, USA) according to the manufacturer’s protocol. Data were analysed as previously described [24]. Briefly, raw sequencing reads were mapped to human genome HG19 applying the TopHat tool (http://ccb.jhu.edu/software/tophat/index.shtml) and differential expression was calculated with the Cufflinks package (http://cole-trapnell-lab.github.io/cufflinks/), using statistically significant cut-offs based on a log_2_ fold-change greater than 1.5 compared to adherent monolayer cultures.

### Single-cell and conventional quantitative RT-PCR

For single-cell sorting, adherent monolayer cultures were enzymatically dissociated with 0.25% Trypsin-EDTA (PAA Laboratories). CSC-enriched subpopulations were collected at 300 x*g* for 5 min, and dead cells were removed with the MACS Dead Cell Removal Kit (Miltenyi Biotec, Bergisch Gladbach, Germany). Cells were stained with FITC-conjugated Annexin V antibody (BD Biosciences, Franklin Lakes, NJ, USA, 1:20) and 0.25 μg 7-AAD (BD Biosciences). Individual Annexin V and 7-AAD negative cells were sorted into 96-well PCR plates (Life Technologies) using a BD FACSAria II (Becton Dickinson) instrument as previously described [25–27]. Reverse transcription, gene-specific preamplification and single-cell qPCR were conducted as described [25]. Single-cell data pre-processing and multivariate analysis were performed with GenEx (version 5.4.3, MultiD, Gothenburg, Sweden) as described [25].

To assess RNA transcript levels of cell populations, RNA was extracted employing the RNeasy Mini Kit (Qiagen). RNA was quantified using the NanoDrop spectrophotometer (Thermo Fisher Scientific, Wilmington, DE, USA). cDNA synthesis was performed with SuperScript III reverse transcriptase (Life Technologies) according to the manufacturer’s instructions. Quantitative RT-PCR was performed in 10 μL reactions using the 2x SYBR GrandMaster Mix (TATAA Biocenter, Gothenburg, Sweden) and was analysed on the CFX384 Touch Real-Time PCR Detection System (Bio-Rad, Hercules, CA, USA) using in-house and commercial qPCR assays (S1 Table).

### Immunoblot

Cell extracts were separated by SDS–polyacrylamide gel electrophoresis and immunoblotted with anti-human HMGCS1 (52 kDa) (HPA036914, Atlas Antibodies, Stockholm, Sweden, 1:1,000) and anti-human actin beta:DyLight^®^680 (42 kDa) (HCA147D680, AbD Serotec, Kidlington, Oxford, UK, 1:10,000) as loading control and analysed using an Odyssey scanner (Licor, Lincoln, NE, USA).

### Tissue microarray

Tumour biopsies from 149 patients with positive lymph-node breast cancer were retrieved from the Department of Pathology at Sahlgrenska University Hospital. The use of patient material for this project involving Sahlgrenska Universitetssjukhuset (Västra Götalandsregionen) and Sahlgrenska Cancer Center (Göteborgs Universitet) was approved by the Regional Research Ethics Committee in Gothenburg (Regionala Etikprövningsnämnden i Göteborg) (DNR: 515–12 and T972-18). All research was performed according to ethical guidelines and written informed consent was obtained from all the participants in the study. Specimens were formalin-fixed and paraffin-embedded. Representative areas of invasive cancer were marked on the original block, and three 1.0-mm tissue cores were collected from each donating tumour block and arranged in a new recipient block using a manual Tissue Arrayer (Beecher Inc., Sun Prairie, WI, USA). Four 0.5 mm sections were cut from HM 355S Rotary Microtome (Thermo Scientific, Waltham, MA, USA) and processed in a standard immunohistochemistry Autostainer (DAKO AutostainerLink 48, Copenhagen, Denmark). Immunohistochemistry was performed on TMA sections using DAKO Autostainer and Envision FLEX+ detection system. Briefly, deparaffinized sections were subjected to antigen retrieval by high pressure cooking and DIVA antigen retrieval, followed by blocking with hydrogen peroxide and incubation with primary antibody against HMGCS1 (Atlas Antibodies, Stockholm, Sweden, 1:500). HMGCS1 expression was assessed using the Allred scoring system. Statistical analysis were computed in SPSS (IBM, version 20, New York, NY, USA)

### Statistical analysis

Data was processed using Excel 2016, GraphPad Prism v7.04 or SPSS (IBM, version 20). Statistical comparisons between two groups were determined using a paired Student's t-test, with statistical significance defined as p < 0.05 (two-tailed). The Mann-Whitney U test was used to analyse the expression of individual genes between two groups in the single-cell experiments, and obtained p-values were Bonferroni-adjusted to correct for multiple testing. Spearman’s correlation was applied to identify significant associations between gene expression in single-cell experiments, and HMGCS1 to various clinical parameters in the tissue microarray. Associations with a p-value of ≤0.05 were considered to be statistically relevant. All experiments were carried out in triplicates unless it was specified.

## Results

### The mevalonate pathway is a key feature of CSC-enrichment in luminal and basal breast cancer subtypes

**Fig 1:**
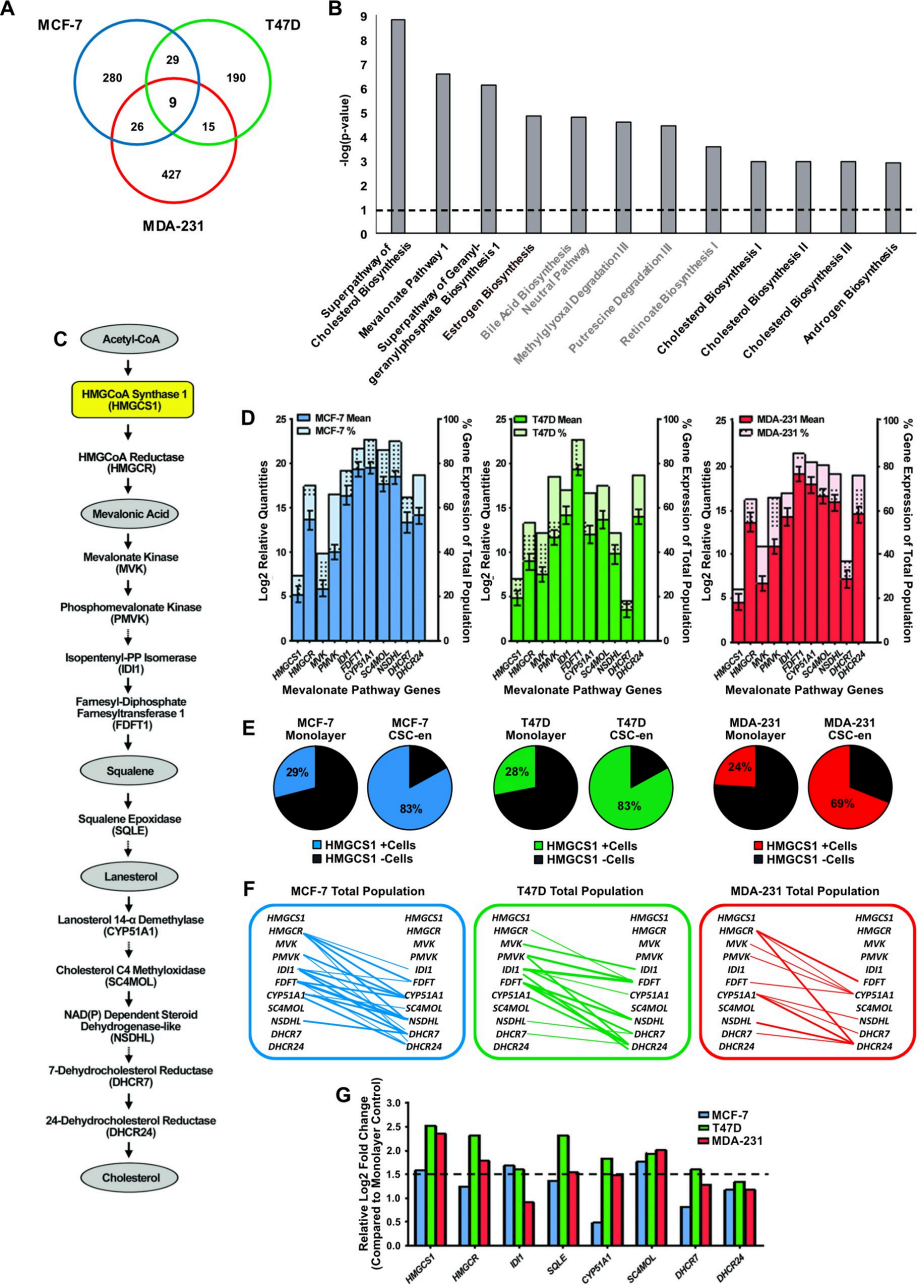
The mevalonate pathway is the most over-represented network in the CSC enriched subpopulations of luminal and basal breast cancer subtypes. **A.** Venn diagram representing the significant overexpressed genes (> 1.5 fold-change) in the 16- hours CSC-enriched cultures compared to individual adherent monolayer cultures for each cell line. Overall analysis of the RNA-sequencing data for MCF-7, T47D and MDA-MB-231 cell lines, identified 344, 243 and 477 genes. **B.** Representation of the pathway-associated networks significantly above the statistical threshold (p<0.001) in the CSC-enriched cell line subpopulations. **C.** Schematic diagram of several mevalonate pathway enzymatic intermediates. **D.** Single-cell expression analysis of mevalonate pathway genes by qPCR, conducted in MCF-7 (blue, n = 76), T47D (green, n = 88) and MDA-MB-231 (MDA-231) (red, n = 83) adherent monolayer cultures. Mean gene expression ±SEM per single cell for each cell line is represented on left y-axis and the percentage of cells expressing a particular gene within the overall single-cell population is represented on the right y-axis. **E.** Percentage of cells expressing *HMGCS1* in adherent monolayer and CSC-enriched (CSC-en) single cell populations, in MCF-7 (blue), T47D (green) and MDA-MB-231 (red) cell lines. **F.** Line graphs depicting single-cell gene associations using Spearman’s correlation to identify significant correlations between mevalonate pathway genes in each cell line monolayer population (p<0.05, ρ≥0.4). Each significant gene correlation is represented by a line and connecting lines are weighted in an increasing manner to represent increasing correlation coefficient values (S5 Table). **G.** Relative gene expression of mevalonate pathway genes in the CSC subpopulations compared to monolayer controls from the original RNA-sequencing data.

Transcriptome-wide gene expression profiling using high-throughput RNA sequencing (RNA273seq) identified distinct genetic signatures, commonly overexpressed in CSC-enriched subpopulations of breast cancer cell lines. Two luminal (MCF-7 and T47D) and one basal (MDA-MB-231) cell lines were cultured as adherent monolayers or using a 16 hour suspension culture system to functionally enrich for CSC-enriched subpopulations [22]. A total of 344 genes and 243 genes were significantly up-regulated in the MCF-7 and T47D CSC-enriched subpopulations, respectively, as well as 477 genes in the MDA-MB-231 subpopulation (Fig 1A and S1 Data). Ingenuity® Pathway Analysis (IPA®, QIAGEN Redwood City, CA, USA) was applied to the 79 genes that were significantly overexpressed in two or more of the CSC-enriched cell line subpopulations (S2 Table). Eight out of the 12 most over-represented pathways (p<0.001) were mevalonate associated networks (Fig 1B and S3 Table).

To determine similarities across the three cell lines, the mean gene expression and the percentage expression of 11 mevalonate genes (*HMGCS1*, *HMGCR*, *MVK*, *PMVK*, *IDI1*, *FDFT1*, *CYP51A1*, *SC4MOL*, *NSDHL*, *DHCR7* and *DHCR24*) in each cell line (MCF-7; n = 76, T47D; n = 88, MDA-MB-231; n = 83) were assessed by single-cell gene expression profiling (Fig 1D). Comparable patterns of expression were observed for *HMGCS1*, *MVK* and *DHCR24* in the three cell lines (S4 Table). *HMGCS1* expression, in particular, showed no significant difference between cell lines and moreover, was specific to a smaller sub-fraction of tumour cells within each monolayer single-cell population. Comparative analysis of *HMGCS1* expression in adherent monolayer culture and in the 16 hour CSC-enriched model showed a significant expansion of *HMGCS1* expressing cells at the single cell level; with a 54% increase in MCF-7 cells, 55% in T47Ds and 45% in MDA-MB-231s following CSC-enrichment (Fig 1E). Spearman’s correlation analysis was applied to further test the strength of association between mevalonate genes in each cell line. MCF-7 cells exhibited 21 significant positive correlations between the mevalonate genes (where p<0.05, ρ≥0.4), whilst the T47D and MDA-MB-231 cells had 13 each (Fig 1F and S5 and S6 Tables). From this analysis, *HMGCS1* was the only gene which commonly lacked any association to the other pathway genes. *HMGCS1*, although an important precursor to the first committed step of the mevalonate pathway, appeared to behave entirely independent from the other pathway members. Furthermore, it was together with *SC4MOL* one of the only nine genes significantly over-expressed in all three CSC-enriched cell lines in the original RNA-seq dataset (Fig 1G and 1A), thus making *HMGCS1*, independent of the mevalonate pathway, an intriguing candidate marker for functional CSC-enrichment, with potential relevance in both luminal and basal subtypes.

### HMGCS1 regulates CSC-enrichment in luminal and basal breast cancer subtypes

**Fig 2:**
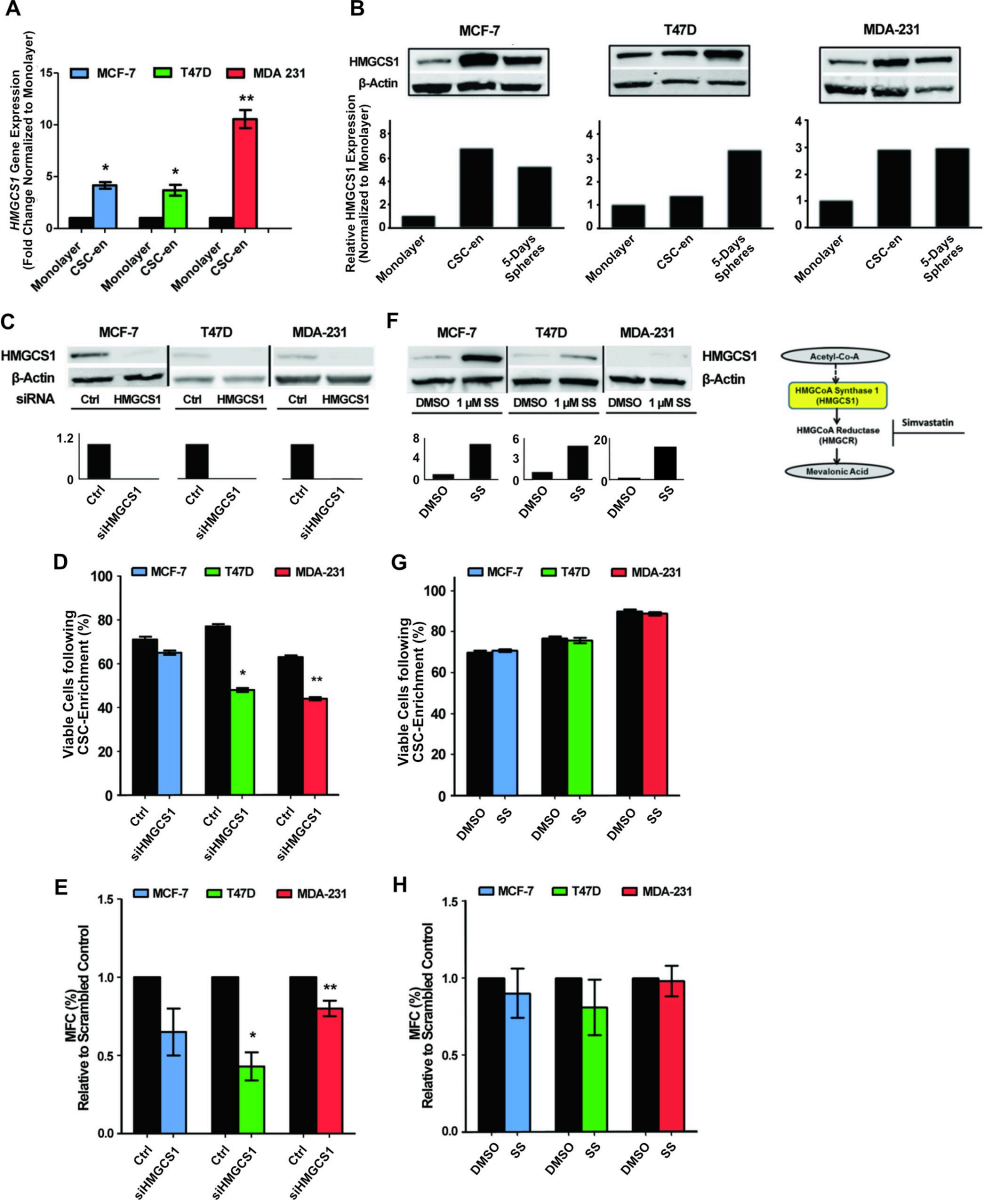
High expression of HMGCS1 was a consistent feature in CSC enriched subpopulations of luminal and basal breast cancer subtypes and, the silencing of *HMGCS1* gene expression reduced the levels of CSC activity. **A.** *HMGCS1* gene expression measured by qPCR in MCF-7, T47D and MDA-MB-231 (MDA-231) cell lines cultured as adherent monolayer or 16 hour CSC-enriched (CSC-en) cultures. *HMGCS1* transcript levels are relative to individual monolayer controls. Mean ±SEM from three biological replicates are shown. **B.** HMGCS1 protein expression in MCF-7, T47D and MDA-MB-231 cell lines cultured as adherent monolayers, 16 hours suspension cultures or 5-days mammosphere cultures. A representative western blot and associated densitometry are shown. **C.** Representative western blot and associated densitometry of MCF-7, T47D and MDA-MB-231 cell lines transfected with *HMGCS1* siRNA confirming the knockdown of HMGCS1 protein expression, relative to the scrambled negative control. β-actin was used as a loading control. **D.** Percentage of viable cells in 16 hours CSC-enriched cultures of MCF-7, T47D and MDA-MB-231 cells transfected with either scrambled negative control or *HMGCS1* siRNA. **E.** Mammosphere forming capacity (MFC) of the three cell lines transfected with either scrambled negative control or *HMGCS1* siRNA, represented as percentage of total cells seeded. **F.** Representative western blot and associated densitometry confirming the increase of HMGCS1 protein in cells treated 48 hours with 1 μM Simvastatin (SS), compared to the control (DMSO) samples for each cell line. **G.** Percentage of viable cells in 16 hours CSC-enriched cultures of MCF-7, T47D and MDA-MB-231 cells treated with either DMSO control or 1 μM Simvastatin. **H.** Mammosphere forming capacity (MFC) of the three cell lines treated with either DMSO control or 1 μM Simvastatin. In all graphs the mean ±SEM of three independent experiments is shown and, the significance level was determined by two-tailed, paired Student’s t-test; *p<0.05, **p<0.01.

Over-expression of *HMGCS1* transcript levels in the three cell lines following 16 hour CSC-enrichment was confirmed using RT-qPCR (Fig 2A), with the most significant up-regulation observed in the basal MDA-MB-231s (10-fold change). Functional expression of HMGCS1 was measured using two separate models of CSC-enrichment; 16 hour suspension culture and also 5 days mammosphere culture. Western blot analysis showed that HMGCS1 protein expression was increased in both enrichment models in all cell lines compared to the adherent monolayer control (Fig 2B). Accompanying densitometry graphs showed the increases in HMGCS1 protein levels were most marked in the MCF-7 and MDA-MB-231 cell lines, whereas the T47D cells showed only a moderate increase after 16 hours compared to a more significant increase in the 5 day mammosphere culture (Fig 2B). Taken together, the data clearly confirms increased HMGCS1 expression is a feature of CSC-enrichment in both luminal and basal functional models. To assess the functional effect of HMGCS1 on the same two models of CSC-enrichment, *HMGCS1* gene expression was effectively silenced in all three cell lines (Fig 2C). Using the initial 16 hour CSC-enrichment model, loss of HMGCS1 expression induced a decrease in the number of viable cells capable of surviving anchorage-independent conditions in all three cell lines. However, the observed decrease in viability only reached statistical significance in the T47D and MDA-MB-231 cells, where 20–30%, respectively, of reduction in viable cells was detected (Fig 2D). The same cells were seeded for 5 days mammosphere cultures and a similar pattern of regulation was observed. Cells lacking HMGCS1 expression formed less mammospheres after 5 days; however the most significant differences were again limited to the T47D and MDA-MB-231 cell lines (Fig 2E). This data clearly shows that in terms of HMGCS1, functional activity does extend to the luminal CSC models as well the basal [16].

To complement and put into perspective, the effect of HMGCS1 on our luminal and basal model system, cells were treated with a standard dose of Simvastatin (1 μM) for 48 hours. Statin therapy targets 3-hydroxy-3-331 methylglutaryl-CoA Reductase (HMGCR), the major rate limiting enzyme within the mevalonate pathway and the direct downstream substrate for HMGCS1 activity. Statin targeting of HMGCR suppresses the entire mevalonate pathway, reducing cholesterol production and protein prenylation [14, 28]. Interestingly, statin treatment increased the levels of HMGCS1 protein in each of the three cell lines (Fig 2F and S1 Fig). A blockade of HMGCR leaves HMGCS1 with no functional substrate and could cause its protein levels to inappropriately accumulate. To highlight the power of HMGCS1 expression alone versus the overall power of the pathway, the same CSC enriched functional models were assessed in Simvastatin-treated cells. Unlike the HMGCS1 knockdown cells, there was no observed reduction in the number of viable cells following the 16 hour CSC-enrichment method (Fig 2G). Also, the 5 days mammosphere CSC-enrichment model, showed no clear reduction in the ability of Simvastatin-treated cells to form functional sphere structures (Fig 2H). The comparable nature of the knockdown and statin treated experiments clearly show that in these models of CSC-enrichment, inhibition of the mevalonate pathway does not have the capacity to influence the survival or activity of CSC enriched subpopulations to the same extent as inhibition of HMGCS1 alone. This data suggests that HMGCS1 ability to enzymatically facilitate anabolic reactions and to initiate the mevalonate pathway is a more effective therapeutic target than the statin targeted, HMGCR.

### HMGCS1 influences the expression of CSC-associated genes in the basal model of breast cancer

**Fig 3:**
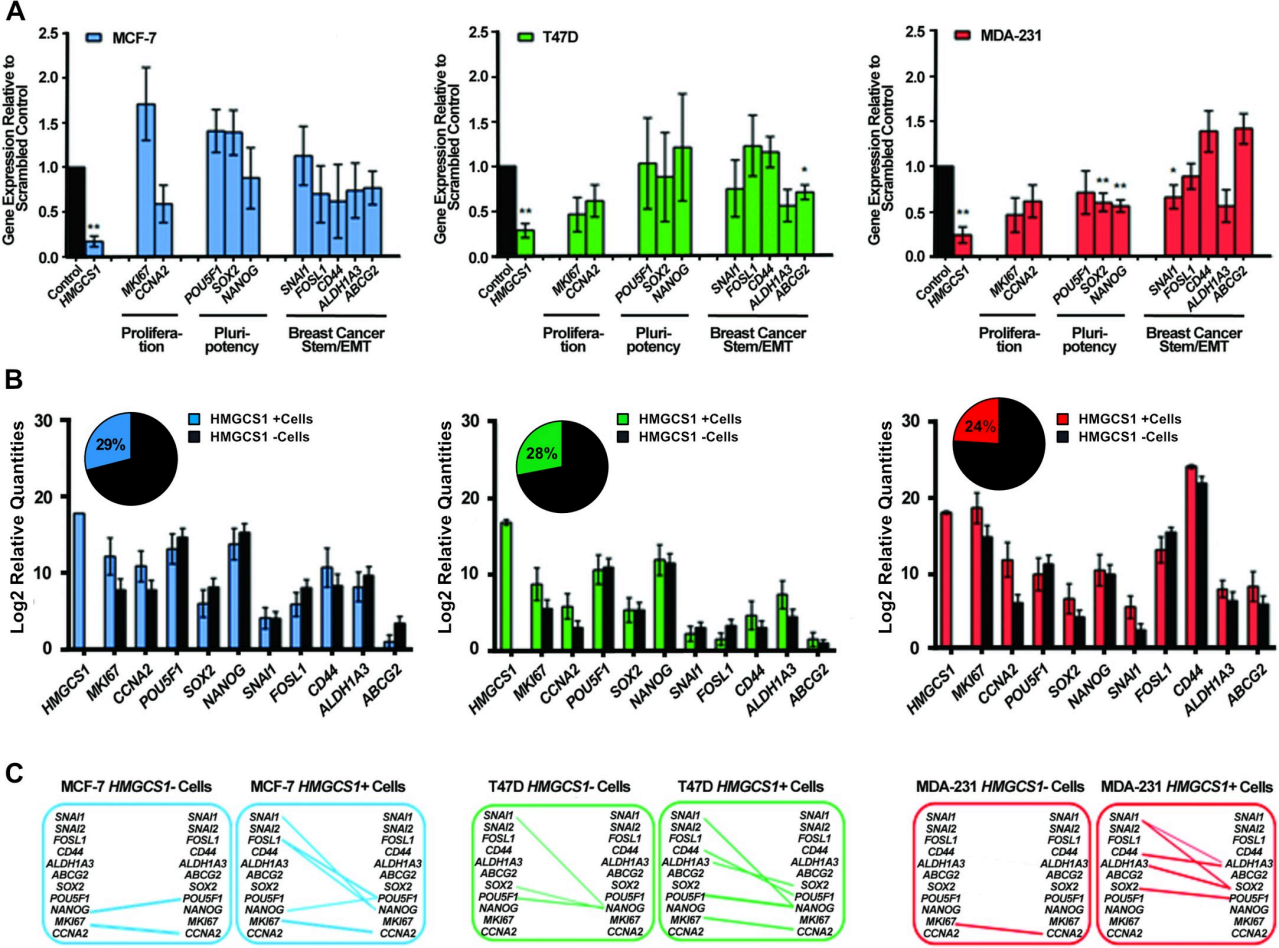
Silencing of *HMGCS1* gene expression reduced the expression of CSC-associated genes in the basal model of breast cancer. **A.** RT-qPCR analysis of the transcript levels of proliferation, pluripotency and EMT/stem-like related genes for MCF-7, T47D and MDA-MB-231 (MDA-231) cells transfected with either scrambled negative control or *HMGCS1* siRNA. Expression levels for each individual gene were normalized to the scrambled negative control for each cell line. Graphs show mean ±SEM, n = 3, *p<0.05, **p<0.01 by two-tailed paired Student’s t-test. **B.** Single-cell gene expression profiling in single cells expressing *HMGCS1* (blue, green, red) versus cells which do not express *HMGCS1* (black), of adherent monolayer cultures for MCF-7 (blue, n = 76), T47D (green, n = 88) and MDA-MB-231 (red, n = 83) cell lines. Mean ±SEM is shown. The Mann-Whitney U test was used to analyse expression of individual genes between two groups and obtained p-values were Bonferroni-adjusted to correct for multiple testing (S6 Table). **C.** Spearman’s correlation analysis was conducted on single-cell populations which did not express *HMGCS1* (MCF-7, n = 54; T47D, n = 63; and MDA-MB-231, n = 62) or expressed *HMGCS1* (MCF-7, n = 22; T47D, n = 25; and MDA-MB-231, n = 21), respectively. Correlations were drawn between the same proliferation, pluripotency and EMT/stem-like genes as analysed in A (S7–S9 Tables). Connecting lines were weighted in an increasing manner to represent increasing correlation coefficient values (p<0.05, ρ≥0.4).

A panel of CSC-associated genes was selected to investigate the transcriptional influence of *HMGCS1* expression within the three cell lines. The selected panel included the proliferative markers, (*MKI67*, *CCNA2*) in addition to the pluripotency markers, (*POU5F1*, *SOX2*, *NANOG*) and a number of prominent and established epithelial-to-mesenchymal-transition (EMT) (*SNAI1*, *FOSL1*) and stem cell markers (*CD44*, *ALDH1A3*, *ABCG2*). Transcript levels were measured in *HMGCS1*-expressing cells and in *HMGCS1*-knockdown cells (Fig 3A). The T47D and MDA-MB-231 cells showed marked reductions for *MKI67* and *CCNA2* in the absence of *HMGCS1* however, neither reached statistical significance. *HMGCS1* expression had no clear effect on the transcriptional regulation of the pluripotency genes in either of the luminal cell lines. With regards to the various stem cell markers, no significant effect was observed in the MCF-7 cell line, whilst the T47Ds asserted a regulatory effect on *ABCG2* expression. Similar to the CSC-enrichment models, *HMGCS1* exhibited much more gene regulatory function in the basal MDA-MB-231 cells. Loss of *HMGCS1* expression in the MDA-MB-231s significantly decreased the transcript levels of *SOX2* and *NANOG* and showed a concomitant loss of *SNAI1* expression. Also *ALDH1A3* expression was markedly reduced but failed to reach statistical significance.

To mimic the HMGCS1 knockdown experiment, the adherent monolayer single cell data was subdivided and analysed in terms of cells expressing *HMGCS1* and cells which did not (Fig 3B). As described in Fig 1E, the *HMGCS1*-expressing cells had remarkably similar frequency in each cell line; 29% of MCF-7s, 28% of T47Ds and 24% of the MDA-MB-231s. The same panel of genes was analysed to assess whether a similar pattern of regulation could be observed at the single-cell level. Numerous trends were observed across the three cell lines, however none reached statistical significance. At the single-cell level HMGCS1-negative cells of all cell lines exhibited a marked reduction of the proliferative genes, *MKI67* and *CCNA2*. For the pluripotency markers, no obvious pattern of regulation was observed among the luminal cell lines, however in the MDA-MB-231 cells, again there was a clear reduction in *SOX2* transcript levels in cells lacking *HMGCS1*. Each cell line also showed a marginal reduction in *CD44* in the *HMGCS1-*negative cells. Among the T47D and MDA-MB-231 *HMGCS1-*negative cells; there was a clear decrease in *ALDH1A3* and *ABCG2* transcript levels. Finally, there was a distinct reduction in *SNAI1* transcript levels in the MDA-MB-231 *HMGCS1-*negative cells; which correlated well with the *HMGCS1* knockdown data. Similar to the CSC-enriched functional models, the more definitive analysis was limited to the more tumourigenic p53-mutated T47Ds and the basal MDA-MB-231s.

Correlation analysis assessed comparative single-cell associations between the same genes in *HMGCS1-*expressing cells and *HMGCS1-*negative cells (Fig 3C and S7–S9 Tables). Interestingly, similar as we reported in previous studies in anoikis-resistant cultures [27], there was a clear increase in the number of associations of CSC-related genes in the *HMGCS1* expressing cells in all cell lines. Different gene associations were exhibited by the different cell lines, with a strong and significant *MKI67* / *CCNA2* association in both luminal *HMGCS1-*expressing cells, while the strongest gene associations present in the MDA-MB-231 *HMGCS1-*expressing cells was between pluripotent markers, *SOX2 / POU5F1* and between the well-known stem markers, *ALDH1A3 / CD44*. The single-cell analytical approach applied to *HMGCS1* gene function confirmed the initial transcriptional observations, although the distinct functionality and molecular interaction of this enzyme when present in differing breast cancer subtypes need further studies.

### HMGCS1 expression correlates with disease aggression and associates with basal tumours in a breast cancer patient cohort

**Fig 4:**
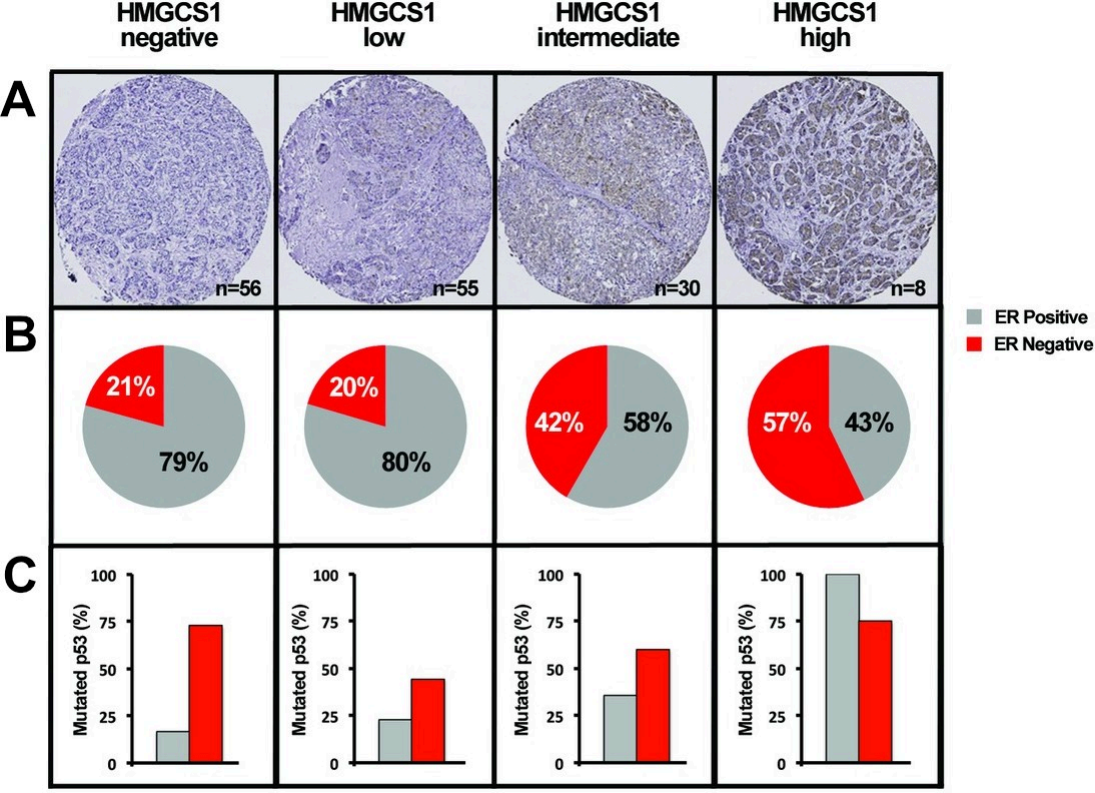
HMGCS1 expression associates with basal tumours in a breast cancer patient cohort. **A.** HMGCS1 expression in a tissue microarray (TMA) from 149 patients with lymph-node positive breast cancer. **B.** Comparative pie charts for each HMGCS1 subgroup (negative, low, intermediate and high) showing the frequency of ERα-positive and ERα-negative tumour subtypes present in each. **C.** Percentage of p53 mutations in ERα-positive and ERα-negative breast cancers for HMGCS1 subgroups.

To confirm the clinical relevance of HMGCS1 expression, a cohort of 149 lymph node positive breast cancer tumours was stained and sub-classified for HMGCS1 protein expression (Fig 4A). Correlation analysis of HMGCS1 expression identified significant positive correlations with tumour grade, p53 mutational status, and hypoxia-inducible factor 1-α (Table 1), all of which are well established features of disease aggression [29–31]. The molecular data has already shown a clear correlative pattern between the functional effects of HMGCS1 and the increased tumourigenicity of the subtypes in which it was assessed, with the most significant regulation consistently observed in the basal MDA-MB-231 cells. The clinical correlations complement these original observations and the specific association of HMGCS1 with a distinctly aggressive disease phenotype. Moreover, *in silico* Kaplan-Meier analysis of a public breast cancer dataset [32] revealed that high expression of HMGCS1 was associated with worse relapse-free survival (RFS) (S2 Fig).

**Table 1:** Clinical associations of HMGCS1 in a lymph node positive breast cancer patient cohort.

	Grade	ER^a^	PR^b^	P53 mutation	HIF1α^c^
***Spearman’s Correlation***	0.326	-0.311	-0.393	0.276	0.256
***Significance (p-value)***	<0.001	<0.001	<0.001	0.001	0.003
***n***	122	129	124	147	133

Spearman’s correlation coefficients of HMGCS1 to various clinical parameters were determined using a tissue microarray of 149 lymph-node positive breast cancer biopsies as described in Fig 4A. Associations with a p-value of ≤0.05 were considered to be statistically relevant. All statistical analysis was computed in SPSS (IBM, version 20).

^a^ER, estrogen receptor α

^b^PR, progesterone receptor

^c^HIF1α, hypoxia inducible factor 1-α

In addition to the positive correlations, HMGCS1 exhibited significant inverse correlations with estrogen receptor α (ER) and progesterone receptor (PR), further supporting HMGCS1 activity in the basal subtypes. Furthermore, there was an obvious increase in the frequency of ER-negative tumours in the HMGCS1-intermediate (42%) and -high (56%) tumours (Fig 4B), as well as an increase in p53 mutation in ERα-positive tumours (Fig 4C). Whilst HMGCS1 is present and functional within luminal subtypes of breast cancer, all the analysis suggests that its effects may be most deliberate and consequential in the basal subtypes.

## Discussion

There is increasing evidences that CSCs influences many key processes involved in breast cancer progression, including tumour initiation, promotion and metastasis [33]. Several studies have also implicated a role for this subpopulation of cancer cells in therapeutic resistance. Developing breast CSCs targeting therapy approaches is therefore of high interest [33]. Despite these efforts, CSC targeting strategies are difficult due to for example molecular heterogeneity between CSCs in different breast cancer molecular subtypes [34]. In this study, we sought to identify common molecular pathways to luminal and basal breast cancers to in part overtake this limitation by applying genome-wide RNA sequencing of functionally enriched CSCs in conjunction with matched adherent cultures for both breast cancer subtypes.

Using this approach, we observed that the mevalonate pathway was accentuated in CSC-enriched populations of both subtypes of breast cancer. Similar overrepresentation of the mevalonate pathway in CSCs has been described earlier but restricted to basal breast cancers [16]. In the basal breast cancer study, gene expression signatures were derived from secondary passages of mammosphere cultures grown for 4–7 days. Those cultures will by definition contain mammosphere-initiating as well as differentiated progenitor cells which might be one of the reasons of failing to find active pathways in luminal CSCs. In our study, we used 16-hours anchorage-independent culture system, and we have previously shown that these suspension cultures will enrich for highly tumourigenic mammosphere-initiating cells featuring a CSC-like phenotype [23].

When detailing the mevalonate pathway, we identified HMGCS1 as a common functional marker for CSCs based on that 1) it was significantly overexpressed and expanded in both 16 hours CSC-enriched cell lines subtypes; 2) its mRNA and protein levels were increased in both enrichment models, 16 hours suspension and 5 days mammosphere cultures, respectively; and 3) transient *HMGCS1* gene-silencing led to reduced survival of cells in anchorage-independent conditions and mammosphere formation capacity. Independently of the mevalonate pathway, this gene was previously identified as a potential candidate involved in tumour stem-like breast cancer cells, using a meta-analysis on combined gene expression profiles from several studies that utilized tumour sphere technology [35]. Moreover, they confirmed that *HMGCS1* gene was up-regulated in MCF-7 derived sphere cells compared to adherent monolayer growing cells.

Although we identified *HMGCS1* gene overexpressed in CSC-enriched cultures from the three cell lines tested, including both luminal and basal subtypes, the degree of upregulation in the CSC population was cell line dependent. The effect of the *HMGCS1* gene-silencing upon the CSC viability and mammosphere formation was more significant in T47D and MDA-MB-231 compared to MCF-7. When the expression of CSC-associated genes was analysed in cells lacking HMGCS1, the three cell lines showed a reduction of proliferation markers whereas the most significant down-regulation in pluripotency (*SOX2*) and EMT markers (*SNAI1* and *CD44*) were observed in MDA-MB-231. The effect of the *HMGCS1* expression in the proliferation markers *MKI67* and *CCNA2* were confirmed by observations in our previous study where the HMGCS1 expressing cells from mammosphere cultures showed higher expression of these two markers than cells lacking it (S3 Fig) [36]. Besides, these results agree with other studies showing that the blockage of the mevalonate pathway by statins suppressed the expression of a common cluster of genes governing the EMT process in triple negative breast cancer cell line MDA-MB-231 [37].

Interestingly, our data correlated HMGCS1 functional activity with increased tumourigenicity within the cell lines, from luminal to basal. MCF-7 has a typical luminal, less aggressive phenotype; T47D cells, although also luminal, harbour a p53 mutation and are more aggressive, whereas MDA-MB-231 cells are highly aggressive and metastatic [38, 39]. Furthermore, we recognized the effect of HMGCS1 on the CSC population, primarily in cell lines harbouring a p53 mutation (T47D (L194F) and MDA-MB-231 (R280K)); and we further observed a specific association between HMGCS1 protein expression and mutated p53 in a cohort of lymph node-positive breast cancer. Dysregulated mevalonate pathway activation has previously been linked to mutated p53 [15, 30]. This regulation require the recruitment of the mutant p53 to the gene promoters trough the interaction with the sterol regulatory element-binding proteins (SREBP), including the *HMGCS1* promoter, which has showed a significant binding by mutant p53 in the vicinity of *HMGCS1* sterol regulatory elements (SRE-1) [15]. About 30% of all breast cancers exhibit mutations in the *TP53* gene, but the frequency differs greatly across distinct molecular subtypes [40, 41]. About 80% of the basal tumours display mutations in the *TP53* gene, whereas only about 15% of luminal A tumours carry a p53 mutation [40, 41]. Furthermore, basal tumours are characterized by high tumour grade and lack of ERα, PR and HER2 expression [42]. In our study, in a cohort of lymph node positive breast cancers, tumour specific HMGCS1 expression was inversely associated with estrogen and progesterone receptors presence, but positively correlated to aggressive disease phenotype. Our data suggests that that therapeutic modulation of the mevalonate pathway may be beneficial for patients presenting p53 gain-of-function mutations and consequential mevalonate pathway hyperactivation, independent of breast cancer subtype.

In agreement with other studies, we observed that Simvastatin treatment led to elevated HMGCS1 protein levels [17, 43]. A statin-dependent upregulation of HMGCS1, together with other genes belonging to the mevalonate pathway, has also been described in multiple myeloma cells [44]. This feedback mechanism has been associated with statin-insensitivity upon statin exposure in leukemic cells [44], and recently also in breast cancer cells [45]. In our study, Simvastatin treatment had no or mild effect in the viability of the CSC-enriched cultures of the three assayed cell lines. Similar marginal effects in T47D and MCF-7 cells following exposure to statins have been described by other groups, meanwhile a high reduction in viability of the triple negative cell line MDA-MB-231 has been reported. [45]. Here, we showed that by using 16-hours CSC-enriched cultures, the MDA-MB-231 CSC-population had an enriched HMGCS1 expression, and that Simvastatin, as in the luminal cell lines MCF-7 and T47D, led to an increase of the HMGCS1 protein in MDA-MB-231 cells. This may explain the discrepancies with other studies. Given the pronounced effect of *HMGCS1* gene-silencing on CSC survival and seemingly mevalonate-pathway-independent regulation of HMGCS1, pharmacological inhibition of HMGCS1 may be a superior approach to specifically target CSC in luminal and basal breast cancer subtypes.

Taken together, the findings of this study assert that a dysregulated metabolic state is a distinct feature of CSC-enriched luminal and basal breast cancer subtypes and that in particular; the enzyme HMGCS1 is a novel and potential general marker of this enrichment. Whilst an in-depth mechanistic study of HMGCS1-mediated transcriptional effects, lies beyond the scope of this article, the evidence strongly suggests, that HMGCS1 has genuine potential as a therapeutic target, especially in aggressive and basal breast cancer populations.

## Supporting information

S1 Fig Replicates confirming the increase of HMGCS1 protein in cells treated 48 hours with 1 μM Simvastatin (SS), compared to the control (DMSO) samples for each cell line. Western blots images and associated densitometry are shown. Supporting information of Fig 2F.
(PDF)

S2 Fig Prediction of the patient outcomes using the *HMGCS1* gene high and low expression in the different cancer subtypes and ER status. Kaplan-Meier analysis was done in http://kmplot.com [32]. Long-rank statistical test was applied and p-value < 0.05 were considered significant. Hazard ratio (HR) is shown.
(PDF)

S3 Fig Single-cell gene expression profiling in single cells expressing low and high *HMGCS1* versus cells which do not express *HMGCS1* (black), of mammosphere culture for MDA-231 Luciferase. Data derived from RNA sequencing experiments in [36]. The *HMGCS1* mean value of the entire population was used as a cutoff for the classification in low/high *HMGCS1* expressing cells. The number of cells in each group with detectable reads (N) and the expression mean value for each gene (expressed in reads per million, RPM) is summarized in the upper table and the graph representation of the means.
(PDF)

S1 Table Detailed qPCR assay information. (DOCX)

S2 Table Genes exhibiting a positive fold change ≥ 1.5 in 16 hours suspension culture compared to adherent monolayer culture. (DOCX)

S3 Table Ingenuity pathway analysis of over-represented pathways in cancer stem cell-enriched breast cancer subpopulations, p ≤ 0.01. (DOCX)

S4 Table Statistical analysis of mevalonate gene expression in adherently grown single-cell populations of MCF-7, T47D and MDA-231 cell lines. (DOCX)

S5 Table Spearman correlation coefficients of representative genes of the mevalonate pathway for MCF-7, T47D and MDA-231 cells. Correlations exhibiting p-values ≤ 0.05 are shown.
(DOCX)

S6 Table Common Spearman’s gene correlations of mevalonate gene expression in breast cancer cell lines at the single-cell level. Correlations coefficients with ρ ≥ 0.4 and corresponding p-values ≤ 0.05.
(DOCX)

S7 Table Spearman’s single-cell gene correlations of proliferation-, pluripotency- and breast cancer stem cell-/EMT-associated genes in MCF-7 single-cells, separated based on the presence of HMGCS1 expression. (DOCX)

S8 Table Spearman’s single-cell gene correlations of proliferation-, pluripotency- and breast cancer stem cell-/EMT-associated genes in T47D single-cells, separated based on the presence of HMGCS1 expression. (DOCX)

S9 Table Spearman’s single-cell gene correlations of proliferation-, pluripotency- and breast cancer stem cell-/EMT-associated genes in MDA-213 single-cells, separated based on the presence of HMGCS1 expression. (DOCX)

S1 Data Complete list of genes with the expression values for each cell line exhibiting a positive fold change > 1.5 in 16 hours suspension culture compared to adherent monolayer culture. (XLSX)

S2 Data (XLSX)

S3 Data (XLSX)

S1 Raw images (PDF)
