# Supplementary figures and images for: The mevalonate precursor enzyme HMGCS1 is a novel marker and key mediator of cancer stem cell enrichment in luminal and basal models of breast cancer
Source: PLoS One. 2020 Jul 21;15(7):e0236187. doi: 10.1371/journal.pone.0236187 (PMC7373278; doi:10.1371/journal.pone.0236187)

**S1 Fig**

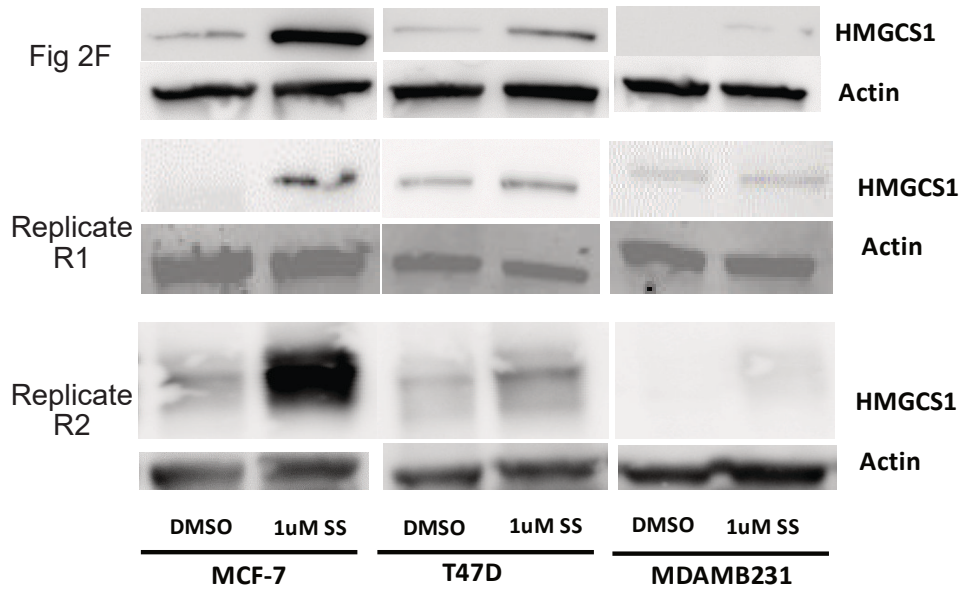

**Fig 2F**

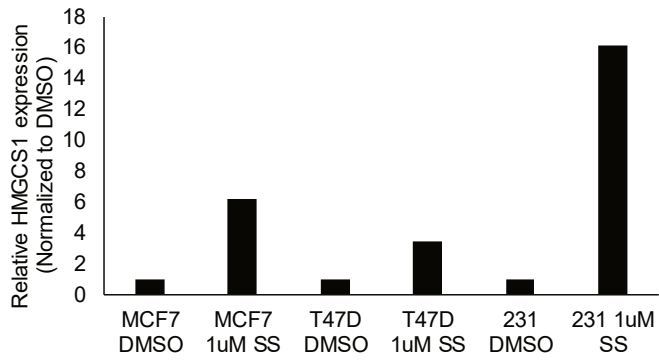

**Replicate R1**

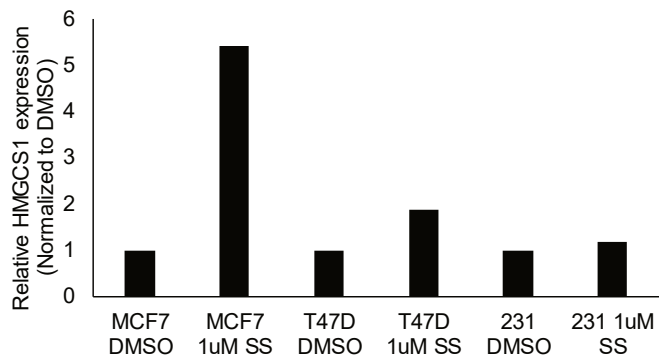

**Replicate R2**

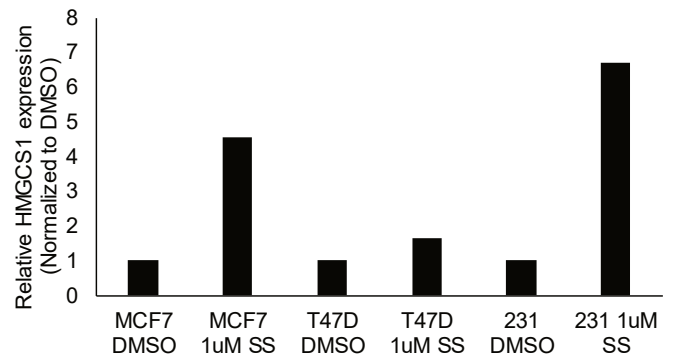

Supplement: S1 Fig — Western blots images and associated densitometry are shown. Supporting information of Fig 2F. (PDF) [file pone.0236187.s001.pdf]

Basal

Luminal A

Luminal B

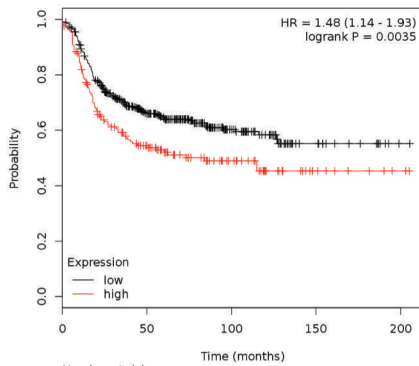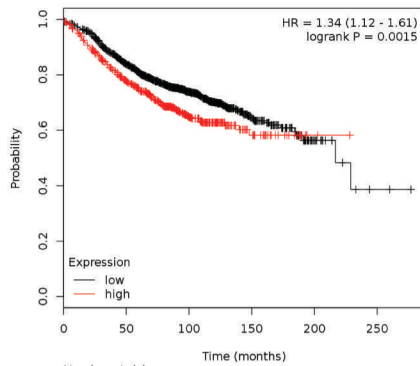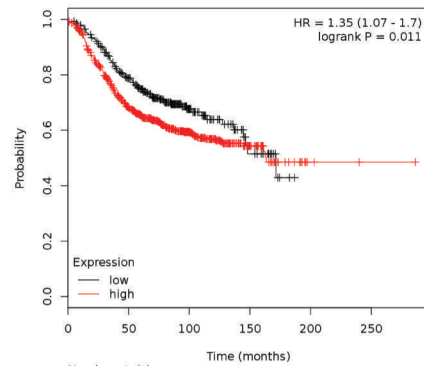ER $\alpha$ -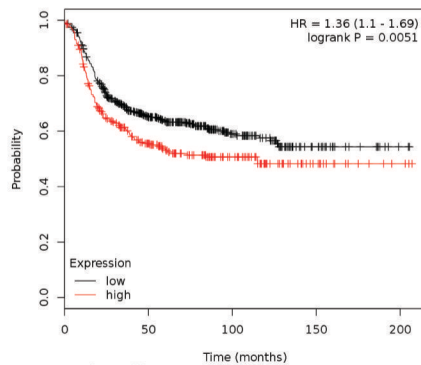ER $\alpha$ +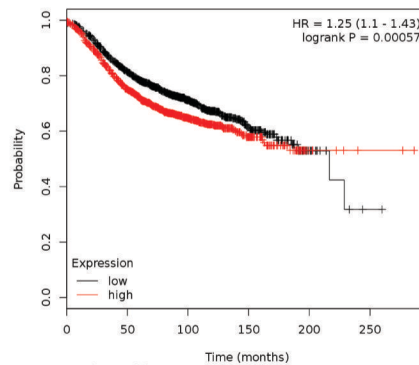

S2 Fig

Supplement: S2 Fig — Kaplan-Meier analysis was done in http://kmplot.com [32]. Long-rank statistical test was applied and p-value < 0.05 were considered significant. Hazard ratio (HR) is shown. (PDF) [file pone.0236187.s002.pdf]

**S3\_Fig**

|               | HMGCS1-negative |         | HMGCS1-Low |         | HMGCS1-High |         |
|---------------|-----------------|---------|------------|---------|-------------|---------|
|               | N               | Mean    | N          | Mean    | N           | Mean    |
| <i>HMGCS1</i> | 8               | 0       | 52         | 13,6277 | 31          | 87,7667 |
| <i>MKI67</i>  | 3               | 88,8909 | 22         | 161,487 | 11          | 133,155 |
| <i>CCNA2</i>  | 3               | 17,6625 | 24         | 90,4588 | 11          | 49,9504 |
| <i>FOSL1</i>  | 6               | 413,681 | 47         | 386,743 | 30          | 531,201 |
| <i>CD44</i>   | 8               | 672,919 | 50         | 411,307 | 29          | 353,491 |

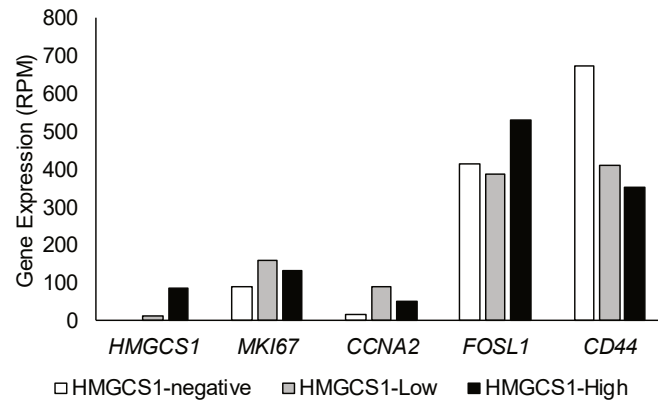

Supplement: S3 Fig — Data derived from RNA sequencing experiments in [36]. The HMGCS1 mean value of the entire population was used as a cutoff for the classification in low/high HMGCS1 expressing cells. The number of cells in each group with detectable reads (N) and the expression mean value for each gene (expressed in reads per million, RPM) is summarized in the upper table and the graph representation of the means. (PDF) [file pone.0236187.s003.pdf]

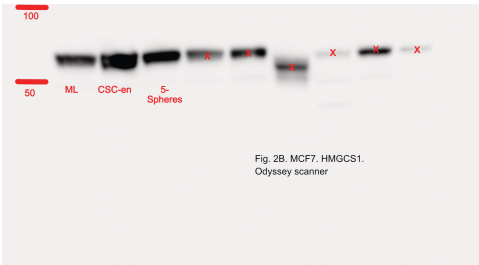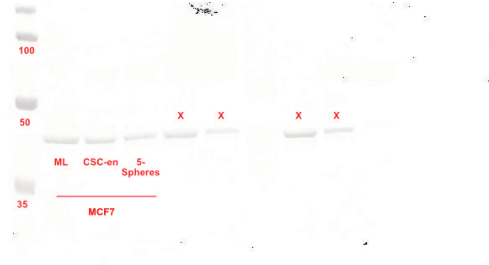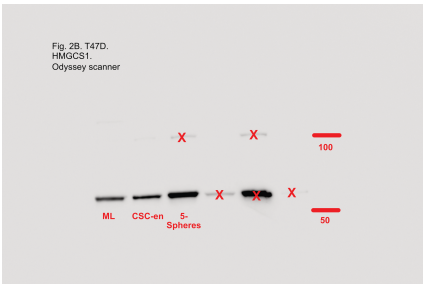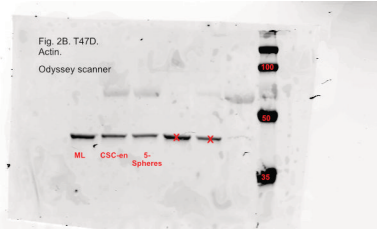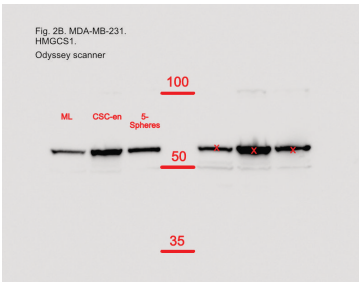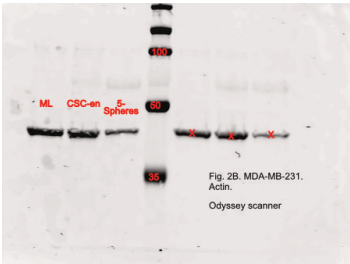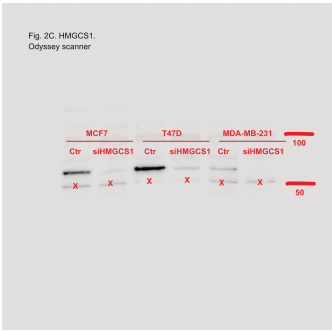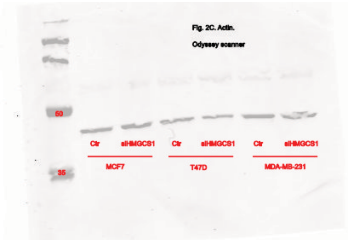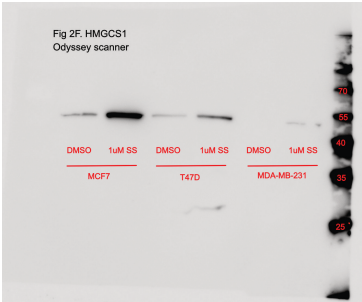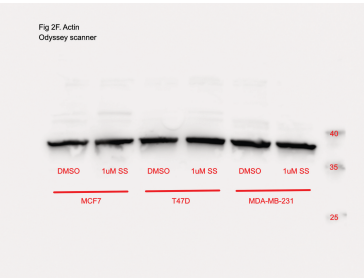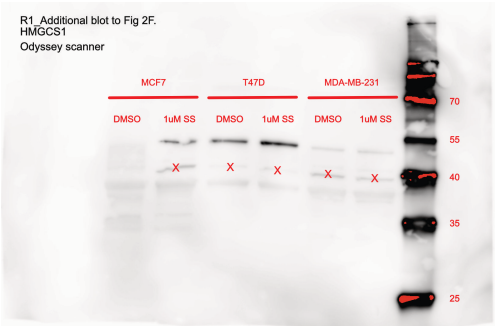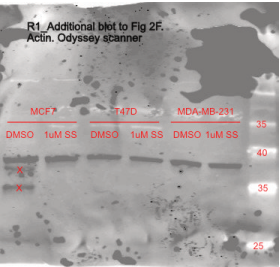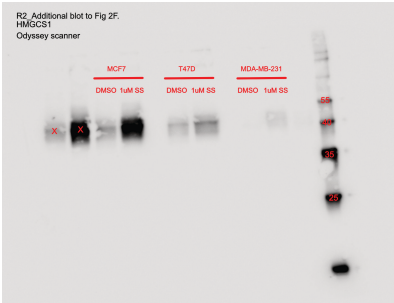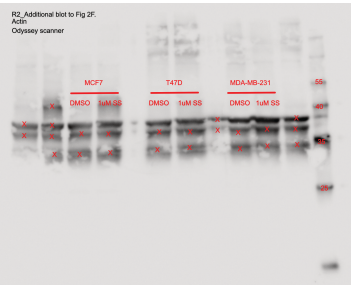

Supplement: S1 Raw images — (PDF) [file pone.0236187.s016.pdf]
