# Supplementary material for: The mevalonate precursor enzyme HMGCS1 is a novel marker and key mediator of cancer stem cell enrichment in luminal and basal models of breast cancer
Source: PLoS One. 2020 Jul 21;15(7):e0236187. doi: 10.1371/journal.pone.0236187 (PMC7373278; doi:10.1371/journal.pone.0236187)
Supplement: S1 Table — (DOCX) [file pone.0236187.s004.docx]

**S1 Table.** Detailed qPCR assay information.

| **Gene Name** | **Ensembl Gene ID** | **Forward Sequence (5’-3)** | **Reverse Sequence (5’-3’)** | **PCR Product Size (bp)** |
| --- | --- | --- | --- | --- |
| ***ABCG2*** | ENSG00000118777 | GGTGGAGGCAAATCTTCGTTA | GAGTGCCCATCACAACATCA | 154 |
| ***ALDH1A3*** | ENSG00000184254 | AAAAAGAGCGAATAGCACCG | GCATAGAGGGCGTTGTAGCA | 132 |
| ***CCNA2*** | ENSG00000145386 | AAGACGAGACGGGTTGC | GGCTGTTTACTGTTTGCTTTCC | 89 |
| ***CD44*** | ENSG00000026508 | GAAGAAGGTGTGGGCAGAAGA | ACCATTTCCTGAGACTTGCTG | 112 |
| ***CYP51A1**** | ENSG00000001630 | CTCTTACCAGGTTGGCTGCCTT | CTTGAGACTGTCTGCGTTTCTGG | 109 |
| ***DHCR7**** | ENSG00000172893 | TCCACAGCCATGTGACCAATGC | CGAAGTGGTCATGGCAGATGTC | 123 |
| ***DHCR24**** | ENSG00000116133 | CAGGAGAACCACTTCGTGGAAG | CCACATGCTTAAAGAACCACGGC | 142 |
| ***FDFT1**** | ENSG00000079459 | TGTGACCTCTGAACAGGAGTGG | GCCCATAGAGTTGGCACGTTCT | 142 |
| ***FOSL1*** | ENSG00000175592 | GCAGGCGGAGACTGACAA | GGGGAAAGGGAGATACAAGG | 219 |
| ***HMGCR**** | ENSG00000113161 | GACGTGAACCTATGCTGGTCAG | GGTATCTGTTTCAGCCACTAAGG | 119 |
| ***HMGCS1**** | ENSG00000112972 | AAGTCACACAAGATGCTACACCG | TCAGCGAAGACATCTGGTGCCA | 115 |
| ***MKI67*** | ENSG00000148773 | TGGGTCTGTTATTGATGAGCC | CATCAGGGTCAGAAGAGAAGC | 188 |
| ***MVK**** | ENSG00000110921 | GGAAAGTGGACCTCAGCTTACC | GCTTCTCCACTTGCTCTGAGGT | 132 |
| ***NANOG*** | ENSG00000111704 | CCTATGCCTGTGATTTGTGG | AAGTGGGTTGTTTGCCTTTG | 166 |
| ***NSDHL**** | ENSG00000147383 | CAGTTTTCCACTGTGCGTCACC | ACGCCCTCAAAGATGACACTGG | 160 |
| ***POU5F1*** | ENSG00000204531 | CGAAAGAGAAAGCGAACCAG | AACCACACTCGGACCACATC | 146 |
| ***PMVK**** | ENSG00000163344 | GCCTTTCGGAAGGACATGATCC | ACTCTCCGTGTGTCACTCACCA | 125 |
| ***SC4MOL**** | ENSG00000052802 | GCTGCCTTTGATTTGTGGAACCT | CTGCACAACCAAAGCATCTTGCC | 113 |
| ***SNAI1*** | ENSG00000124216 | TAATCCAGAGTTTACCTTCCAGCA | AGCCTTTCCCACTGTCCTCA | 224 |
| ***SOX2*** | ENSG00000181449 | ACACCAATCCCATCCACACT | CCTCCCCAGGTTTTCTCTGT | 117 |

*qPCR assays were purchased from Origene Technologies Inc., Rockville, MD, USA; all other qPCR assays were designed in-house using Primer-BLAST (Ye *et al*., 2012).
