# Supplementary material for: The mevalonate precursor enzyme HMGCS1 is a novel marker and key mediator of cancer stem cell enrichment in luminal and basal models of breast cancer
Source: PLoS One. 2020 Jul 21;15(7):e0236187. doi: 10.1371/journal.pone.0236187 (PMC7373278; doi:10.1371/journal.pone.0236187)
Supplement: S2 Table — (DOCX) [file pone.0236187.s005.docx]

**S2 Table.** Genes exhibiting a positive fold change ≥1.5 in 16 hours suspension culture compared to adherent monolayer culture.

| **All Cell Lines** | **MCF-7 and T47D** | **MCF-7 and MDA-231** | **T47D and MDA-231** |
| --- | --- | --- | --- |
| *AQP3* | *ABLIM3* | *AGR2* | *AHNAK2* |
| *CYP1A1* | *AGPAT9* | *AKR1B10* | *ARHGEF37* |
| *FAM83A* | *ANXA1* | *AKR1C1* | *BIRC3* |
| *HMGCS1* | *ATP1A1OS* | *AKR1C2* | *BMF* |
| *HMOX1* | *C9orf152* | *AKR1C3* | *C5orf4* |
| *KRT16* | *CAPN8* | *AKR1C4* | *COL6A1* |
| *SC4MOL* | *DAPP1* | *ALDH3A1* | *DPYSL2* |
| *TRIM29* | *EGLN3* | *CYP1B1* | *HMGCR* |
| *VIPR1* | *EMP1* | *DYNC1I1* | *MAN1A1* |
|  | *ERRFI1* | *FAM46C* | *NFKBIA* |
|  | *FLRT3* | *GBP2* | *PLLP* |
|  | *FOSL1* | *HSPA6* | *SERPINA3* |
|  | *GCLM* | *ITGA10* | *SLCO4A1* |
|  | *GDPD5* | *KRT86* | *SQLE* |
|  | *IDI1* | *MAOB* | *TSC22D3* |
|  | *LMO7* | *METTL7B* |  |
|  | *MVD* | *P2RY6* |  |
|  | *MYEOV* | *PANX2* |  |
|  | *PHLDA2* | *PARM1* |  |
|  | *PYGB* | *PLEKHA4* |  |
|  | *S100A10* | *RNF224* |  |
|  | *SAT1* | *SLC15A3* |  |
|  | *SLC25A18* | *SLC44A3* |  |
|  | *SLC7A8* | *SLCO4C1* |  |
|  | *SQRDL* | *TIMP4* |  |
|  | *ST3GAL1* | *TRIB2* |  |
|  | *TMEM63C* |  |  |
|  | *TXNRD1* |  |  |
|  | *XKRX* |  |  |
