# Supplementary material for: The mevalonate precursor enzyme HMGCS1 is a novel marker and key mediator of cancer stem cell enrichment in luminal and basal models of breast cancer
Source: PLoS One. 2020 Jul 21;15(7):e0236187. doi: 10.1371/journal.pone.0236187 (PMC7373278; doi:10.1371/journal.pone.0236187)
Supplement: S3 Table — (DOCX) [file pone.0236187.s006.docx]

**S3 Table.** Ingenuity pathway analysis of over-represented pathways in cancer stem cell-enriched breast cancer subpopulations, p≤0.01.

| **Ingenuity Canonical Pathways** | **p-value** |
| --- | --- |
| Superpathway of Cholesterol Biosynthesis | 1.62×10^-09^ |
| Mevalonate Pathway I | 2.88×10^-07^ |
| Superpathway of Geranylgeranyldiphosphate Biosynthesis I (via Mevalonate) | 7.94×10^-07^ |
| Estrogen Biosynthesis | 1.45×10^-05^ |
| Bile Acid Biosynthesis, Neutral Pathway | 1.58×10^-05^ |
| Methylglyoxal Degradation III | 2.51×10^-05^ |
| Putrecine Degradation III | 3.80×10^-05^ |
| Retinoate Biosynthesis I | 2.63×10^-04^ |
| Cholesterol Biosynthesis I | 1.15×10^-03^ |
| Cholesterol Biosynthesis II (via 24,25-dihydrolanosterol) | 1.15×10^-03^ |
| Cholesterol Biosynthesis III (via Desmosterol) | 1.15×10^-03^ |
| Androgen Biosynthesis | 1.35×10^-03^ |
