# Supplementary material for: The mevalonate precursor enzyme HMGCS1 is a novel marker and key mediator of cancer stem cell enrichment in luminal and basal models of breast cancer
Source: PLoS One. 2020 Jul 21;15(7):e0236187. doi: 10.1371/journal.pone.0236187 (PMC7373278; doi:10.1371/journal.pone.0236187)
Supplement: S4 Table — (DOCX) [file pone.0236187.s007.docx]

**S4 Table.** Statistical analysis of mevalonate gene expression in adherently grown single-cell populations of MCF-7, T47D and MDA-231 cell lines.

| **Gene** | **MCF-7 and T47D** | **MDA-MB-231** | **T47D and MDA-MB-231** |
| --- | --- | --- | --- |
| *HMGCS1* | 7.35×10^-01^ | 7.92×10^-01^ | 9.82×10^-01^ |
| *HMGCR* | 3.09×10^-05^ | 2.87×10^-02^ | 2.29×10^-06^ |
| *MVK* | 2.00×10^-01^ | 5.88×10^-01^ | 5.12×10^-01^ |
| *PMVK* | 4.46×10^-02^ | 1.21×10^-01^ | 5.21×10^-01^ |
| *IDI1* | 1.55×10^-01^ | 3.80×10^-03^ | 2.93×10^-01^ |
| *FDFT1* | 2.68×10^-03^ | 5.86×10^-01^ | 1.04×10^-02^ |
| *CYP51A1* | <1.00×10^-08^ | 4.62×10^-01^ | 3.25×10^-08^ |
| *SC4MOL* | 3.84×10^-07^ | 2.02×10^-03^ | 1.13×10^-02^ |
| *NSDHL* | <1.00×10^-08^ | 6.79×10^-03^ | 5.13×10^-04^ |
| *DHCR7* | <1.00×10^-08^ | 4.17×10^-05^ | 2.17×10^-02^ |
| *DHCR24* | 6.60×10^-01^ | 8.25×10^-02^ | 1.36×10^-01^ |
