# Supplementary material for: The mevalonate precursor enzyme HMGCS1 is a novel marker and key mediator of cancer stem cell enrichment in luminal and basal models of breast cancer
Source: PLoS One. 2020 Jul 21;15(7):e0236187. doi: 10.1371/journal.pone.0236187 (PMC7373278; doi:10.1371/journal.pone.0236187)
Supplement: S5 Table — Correlations exhibiting p-values ≤ 0.05 are shown. (DOCX) [file pone.0236187.s008.docx]

**S5 Table.** Spearman correlation coefficients of representative genes of the mevalonate pathway for MCF-7, T47D and MDA-231 cells. Correlations exhibiting p-values ≤0.05 are shown.

| **MCF-7 Total Population** | | | **T47D Total Population** | | | **MDA-MB-231 Total Population** | | |
| --- | --- | --- | --- | --- | --- | --- | --- | --- |
| **Gene 1** | **Gene 2** | **Spearman's ρ** | **Gene 1** | **Gene 2** | **Spearman's ρ** | **Gene 1** | **Gene 2** | **Spearman's ρ** |
| *FDFT1* | *NSDHL* | 0.71 | *IDI1* | *FDFT1* | 0.71 | *NSDHL* | *DHCR24* | 0.54 |
| *HMGCR* | *CYP51A1* | 0.53 | *FDFT1* | *NSDHL* | 0.62 | *CYP51A1* | *DHCR24* | 0.53 |
| *PMVK* | *CYP51A1* | 0.52 | *IDI1* | *NSDHL* | 0.57 | *FDFT1* | *NSDHL* | 0.50 |
| *HMGCR* | *FDFT1* | 0.51 | *FDFT1* | *DHCR24* | 0.56 | *HMGCR* | *DHCR24* | 0.49 |
| *IDI1* | *FDFT1* | 0.51 | *PMVK* | *DHCR24* | 0.48 | *HMGCR* | *FDFT1* | 0.48 |
| *IDI1* | *DHCR7* | 0.51 | *MVK* | *IDI1* | 0.47 | *HMGCR* | *CYP51A1* | 0.44 |
| *CYP51A1* | *NSDHL* | 0.51 | *CYP51A1* | *DHCR24* | 0.46 | *DHCR7* | *DHCR24* | 0.44 |
| *CYP51A1* | *DHCR7* | 0.51 | *IDI1* | *DHCR24* | 0.45 | *MVK* | *CYP51A1* | 0.43 |
| *FDFT1* | *DHCR7* | 0.49 | *FDFT1* | *DHCR7* | 0.45 | *FDFT1* | *DHCR24* | 0.42 |
| *CYP51A1* | *DHCR24* | 0.49 | *NSDHL* | *DHCR7* | 0.44 | *CYP51A1* | *NSDHL* | 0.42 |
| *IDI1* | *NSDHL* | 0.48 | *DHCR7* | *DHCR24* | 0.43 | *PMVK* | *CYP51A1* | 0.41 |
| *HMGCR* | *NSDHL* | 0.47 | *HMGCR* | *FDFT1* | 0.42 | *FDFT1* | *CYP51A1* | 0.41 |
| *HMGCR* | *DHCR7* | 0.47 | *IDI1* | *CYP51A1* | 0.41 | *CYP51A1* | *DHCR7* | 0.41 |
| *IDI1* | *CYP51A1* | 0.47 |  |  |  |  |  |  |
| *FDFT1* | *CYP51A1* | 0.45 |  |  |  |  |  |  |
| *NSDHL* | *DHCR7* | 0.45 |  |  |  |  |  |  |
| *HMGCR* | *DHCR24* | 0.44 |  |  |  |  |  |  |
| *CYP51A1* | *SC4MOL* | 0.44 |  |  |  |  |  |  |
| *FDFT1* | *DHCR24* | 0.43 |  |  |  |  |  |  |
| *HMGCR* | *IDI1* | 0.42 |  |  |  |  |  |  |
| *PMVK* | *DHCR7* | 0.40 |  |  |  |  |  |  |
