# Supplementary material for: The mevalonate precursor enzyme HMGCS1 is a novel marker and key mediator of cancer stem cell enrichment in luminal and basal models of breast cancer
Source: PLoS One. 2020 Jul 21;15(7):e0236187. doi: 10.1371/journal.pone.0236187 (PMC7373278; doi:10.1371/journal.pone.0236187)
Supplement: S6 Table — Correlations coefficients with ρ ≥ 0.4 and corresponding p-values ≤ 0.05. (DOCX) [file pone.0236187.s009.docx]

**S6 Table.** Common Spearman’s gene correlations of mevalonate gene expression in breast cancer cell lines at the single-cell level. Correlations coefficients with ρ≥0.4 and corresponding p-values ≤0.05.

| **All Cell Lines** | | **MCF-7 and T47D** | | **MCF-7 and MDA-MB-231** | **T47D and MDA-MB-231** | |
| --- | --- | --- | --- | --- | --- | --- |
| *FDFT1 - DHCR24* | | *NSDHL - DHCR7* | | *HMGCR - DHCR24* | *DHCR7 - DHCR24* | |
| *CYP51A1 - DHCR24* | | *IDI1 - CYP51A1* | | *FDFT1 - CYP51A1* | *FDFT1 - DHCR24* | |
| *HMGCR - FDFT1* | | *IDI1 - NSDHL* | | *CYP51A1 - NSDHL* |  |  |
| *FDFT1 - NSDHL* | | *FDFT1 - DHCR7* | | *CYP51A1 - DHCR7* |  |  |
|  |  | *IDI1 - FDFT1* | | *PMVK - CYP51A1* |  |  |
|  |  |  |  | *HMGCR - CYP51A1* |  |  |
