# Supplementary material for: The mevalonate precursor enzyme HMGCS1 is a novel marker and key mediator of cancer stem cell enrichment in luminal and basal models of breast cancer
Source: PLoS One. 2020 Jul 21;15(7):e0236187. doi: 10.1371/journal.pone.0236187 (PMC7373278; doi:10.1371/journal.pone.0236187)
Supplement: S7 Table — (DOCX) [file pone.0236187.s010.docx]

**S7 Table.** Spearman’s single-cell gene correlations of proliferation-, pluripotency- and breast cancer stem cell-/EMT-associated genes in MCF-7 single-cells, separated based on the presence of *HMGCS1* expression.

| **Gene Correlation** | **Spearman’s ρ** |
| --- | --- |
| **MCF-7 *HMGCS1* Negative Cells** | |
| *MKI67 - CCNA2* | 0.83 |
| *POU5F1 - NANOG* | 0.74 |
| **MCF-7 *HMGCS1* Expressing Cells** | |
| *MKI67 - CCNA2* | 0.68 |
| *FOSL1 - POU5F1* | 0.59 |
| *NANOG - POU5F1* | 0.53 |
| *FOSL1 - NANOG* | 0.53 |
| *SNAI1 - NANOG* | 0.47 |
