# Supplementary material for: The mevalonate precursor enzyme HMGCS1 is a novel marker and key mediator of cancer stem cell enrichment in luminal and basal models of breast cancer
Source: PLoS One. 2020 Jul 21;15(7):e0236187. doi: 10.1371/journal.pone.0236187 (PMC7373278; doi:10.1371/journal.pone.0236187)
Supplement: S8 Table — (DOCX) [file pone.0236187.s011.docx]

**S8 Table.** Spearman’s single-cell gene correlations of proliferation-, pluripotency- and breast cancer stem cell-/EMT-associated genes in T47D single-cells, separated based on the presence of *HMGCS1* expression.

| **Gene Correlation** | **Spearman’s ρ** |
| --- | --- |
| **T47D *HMGCS1* Negative Cells** | |
| *POU5F1 - NANOG* | 0.54 |
| *SNAI1 - NANOG* | 0.42 |
| *SOX2 - NANOG* | 0.42 |
| **T47D *HMGCS1* Expressing Cells** | |
| *POU5F1 - NANOG* | 0.73 |
| *MKI67 - CCNA2* | 0.63 |
| *SNAI1 - NANOG* | 0.45 |
| *CD44 - POU5F1* | 0.45 |
| *ALDH1A3 - SOX2* | 0.45 |
