# Supplementary material for: The mevalonate precursor enzyme HMGCS1 is a novel marker and key mediator of cancer stem cell enrichment in luminal and basal models of breast cancer
Source: PLoS One. 2020 Jul 21;15(7):e0236187. doi: 10.1371/journal.pone.0236187 (PMC7373278; doi:10.1371/journal.pone.0236187)
Supplement: S9 Table — (DOCX) [file pone.0236187.s012.docx]

**S9 Table.** Spearman’s single-cell gene correlations of proliferation-, pluripotency- and breast cancer stem cell-/EMT-associated genes in MDA-213 single-cells, separated based on the presence of *HMGCS1* expression.

| **Gene Correlation** | | **Spearman’s ρ** |
| --- | --- | --- |
| **MDA-231 HMGCS1 Negative Cells** | | |
| *MKI67* | *CCNA2* | 0.57 |
| **MDA-231 HMGCS1 Expressing Cells** | | |
| *CD44* | *ALDH1A3* | 0.59 |
| *SOX2* | *POU5F1* | 0.59 |
| *SNAI1* | *SOX2* | 0.46 |
| *SNAI1* | *ALDH1A3* | 0.40 |
